# Wave Glider Monitoring of Sediment Transport and Dredge Plumes in a Shallow Marine Sandbank Environment

**DOI:** 10.1371/journal.pone.0128948

**Published:** 2015-06-12

**Authors:** Vera Van Lancker, Matthias Baeye

**Affiliations:** Operational Directorate Natural Environment, Royal Belgian Institute of Natural Sciences, Brussels, Belgium; Centro de Investigacion Cientifica y Educacion Superior de Ensenada, MEXICO

## Abstract

As human pressure on the marine environment increases, safeguarding healthy and productive seas increasingly necessitates integrated, time- and cost-effective environmental monitoring. Employment of a Wave Glider proved very useful for the study of sediment transport in a shallow sandbank area in the Belgian part of the North Sea. During 22 days, data on surface and water-column currents and turbidity were recorded along 39 loops around an aggregate-extraction site. Correlation with wave and tidal-amplitude data allowed the quantification of current- and wave-induced advection and resuspension, important background information to assess dredging impacts. Important anomalies in suspended particulate matter concentrations in the water column suggested dredging-induced overflow of sediments in the near field (i.e., dynamic plume), and settling of finer-grained material in the far field (i.e., passive plume). Capturing the latter is a successful outcome to this experiment, since the location of dispersion and settling of a passive plume is highly dependent on the ruling hydro-meteorological conditions and thus difficult to predict. Deposition of the observed sediment plumes may cause habitat changes in the long-term.

## Introduction

To ensure sustainable development of the marine environment, international agreements and environmental legislation call for the monitoring of a range of biotic and abiotic parameters [1,2]. In Europe, the Marine Strategy Framework Directive (MSFD, 2008/56/EC) requires Member States to demonstrate good environmental status of their marine environments by 2020. All elements that make up the ecosystem (physical, chemical and biological variables) and all human activities need consideration, calling for inclusion of functional and ecosystem-based approaches in monitoring programmes [3,4,5]. As such, there is a necessary move from ‘station-oriented monitoring’ to ‘basin or system-oriented monitoring’, in combination with specific ‘cause—effect’ studies [6].

Traditionally, the status of the marine environment is monitored using ships, allowing for synchronous measurements of air, water column and seabed properties [7]. Both station and transect monitoring can be performed, with increasing possibilities when also ships of opportunity, such as ferries, are equipped with instrumentation [8]. Additionally, ships allow, in a most practical way, repetitive mapping of the water column and the seabed, providing unprecedented spatial detail of physical and biological features over vast areas [9,10,11,12]. To obtain long time series and/or higher temporal resolutions of some parameters, the use of moorings and/or multi-sensor benthic landers are required [13,14,15,16]. Expanding on these possibilities, coastal and seafloor observatories most often guarantee a long-term commitment to acquire continuous time series [17,18,19]. However, natural and man-made changes to the marine environment need measurements at different time- and space scales of which the magnitude and extent is often unpredictable [20]. This complicates survey planning, as also the choice of the optimal location of moorings and landers.

Therefore, we assessed the use of an unmanned surface vehicle (USV) as an alternative approach to monitoring. Since their development in the ‘90s [21], USVs have been increasingly deployed, though mostly for surveying along long distances (e.g., crossing the Pacific [22]; southern North Sea [23]). Together with gliders [24,25], as well as autonomous underwater vehicles (AUV) [26,27,28], these new technologies widely increase the potential of environmental monitoring, and of impact assessments in particular.

The USV used in this study is a Liquid Robotics ‘Wave Glider’ [29]. From April 15^th^ to May 6^th^ 2013 (Day of Year (DoY) 105–126), this USV was deployed, for the first time, in a shallow sandbank environment of 8 to 40 m deep water with surface currents of more than 1 ms^-1^. The Belgian part of the North Sea, one of world’s busiest sea areas, proved to be highly challenging for the Wave Glider and its pilots. The USV was fitted with current and turbidity sensors suitable for assessing the effects of marine aggregate extraction, needed ultimately to recommend more sustainable exploitation practices [30]. Scientific aims were (1) collection of a continuous time series on the natural variability of advection and resuspension (‘background conditions’) during a neap-spring cycle, and (2) detection of turbidity plumes created by the dredging activity.

In relation to marine aggregate extraction, one can expect three types of dredge plumes, each having a typical behaviour [31]: (1) a surface plume dispersing away from the vessel (i.e., trailer suction hopper dredger); (2) a dynamic plume, representing the coarser part of the initial plume, and descending in the near field; and (3) a passive plume, bringing together the finest fractions from the surface and dynamic plumes, and from a near-bed plume caused by the draghead. The passive plume can easily extend several km from the vessel [31,32,33]. Research on the transport and fate of the released fine sediments requires a suite of techniques and instruments that can be used to generate long time series over extended areas, and thus increase the chance to measure local effects at locations that are difficult to predict [34,35].

This paper provides the complete framework of the Wave Glider deployment, including the mission plan and sensors used, and interprets the data in a marine aggregate-extraction and sandbank morphodynamics’ context. On the basis of this analytical work, recommendations are given on survey designs optimising future environmental monitoring of human impacts in the shallow-marine environment. Applications are wide-spread, especially when extensive and long spatio-temporal time series are needed (i.e., plumes in river mouths and estuaries), or where the use of small surface vessels are considered too dangerous: e.g., for measuring hydrothermal discharges in shallow water or for assessing water turbidity effects over lava flows entering the ocean.

## Study Area

**Fig 1:**
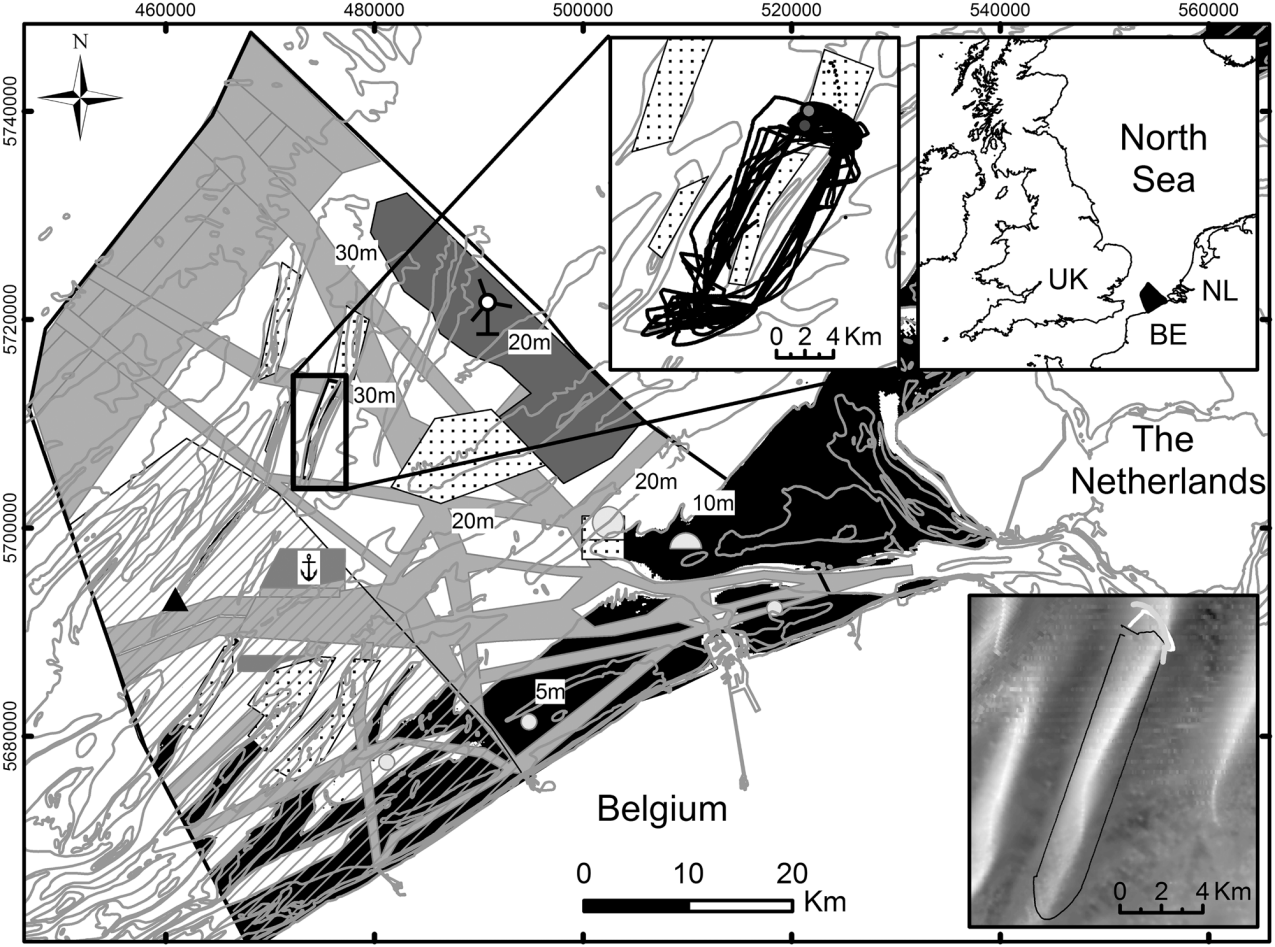
Belgian part of the North Sea with the location of the Wave Glider experiment. Left inset shows the detailed trajectory of 39 laps (22 days) around a marine aggregate concession zone (dotted area). A Habitat Directive area (hatched) is present as close as 2.5 km from the southernmost extraction sector. The Wave Glider could only operate outside of navigation routes (light grey), in areas deeper than -10 m (non-black), and outside a safety buffer of 1 km around major human activities (e.g., wind-farm area, darkest grey; anchor zone, dark grey). Also shown is the location of Flanders Hydrography’s hydro-meteo pole MOW7 (triangle) at the Westhinder sandbank, close to which a Wavec buoy measures wave parameters. Lower right inset is a digital terrain model of the area of the experiment. Superimposed is a typical Wave Glider trajectory, as also profile locations. Main bathymetric contours (Mean Lowest Low Water, Spring) have been labelled.

The monitoring of advection and resuspension and the dynamics of dredge plumes was investigated in the Hinder Banks area, a sandbank complex located 40 km offshore in the Belgian part of the North Sea (BPNS). Depths of the sandbank crests range from -8 m to -30 m mean lowest low water at Spring (MLLWS); they are superimposed with a hierarchy of dune forms, commonly more than 6 m in height. The lows in between the sandbanks reach depths up to -40 m. At present, extraction of aggregates takes place mainly on the Oosthinder sandbank (Fig 1). Here, medium- to coarse sands dominate with less than 1% of silt-clay, however, locally higher percentages have been measured [36]. Near-bottom tidal currents reach up to 1 ms^-1^; for 2011–2013, significant wave heights exceeded 1 m for approximately 44% of the time.

Over a 10-yr period, intensive extraction of marine aggregates (up to 2.9 million m³ over 3 months) is allowed in this area, with a maximum of 35 million m³ over a period of 10 years. The largest vessels can extract 12500 m³ per run. For the entire BPNS, yearly volumes recently surpassed 3 million m³, the majority of which has been extracted using vessels with an individual capacity of 1500 m³. The intensive extraction is new practice in the BPNS and the environmental impact is yet to be determined. South of the Hinder Banks concession, a Habitat Directive area is present, hosting ecologically valuable gravel beds [37]. To prevent degradation of these beds, it is critical to assess the effect of multiple and frequent deposition events related to dredge plumes.

## Materials and Methods

### Ethics statement

The Wave Glider‘s mission plan accounted for a safety buffer of 1 km around the delineation of the marine aggregate sector (Ministerial Decree 2010-12-24/03). Flemish Authorities, Agency Maritime Services and Coast (MDK), Maritime Rescue and Coordination Centre (MRCC) and the Coast Guard granted permissions for the experiment. MDK’s Coast department, commissioner of the marine aggregate extraction activities was notified of the experiment. The field studies had no impact on endangered or protected species.

### Wave Glider

#### Platform

The Wave Glider of Liquid Robotics is a commercially available USV, measuring simultaneously in air and water. Its propulsion is based on the conversion of wave motion into thrust and the vehicle utilises solar power to feed its instruments (e.g., for navigation and measurements). This technology is highly favourable where employment endurance is of paramount importance [38]. Long-term integrated data series can thus be captured at the same or reduced cost as from ships and using buoys.

**Fig 2:**
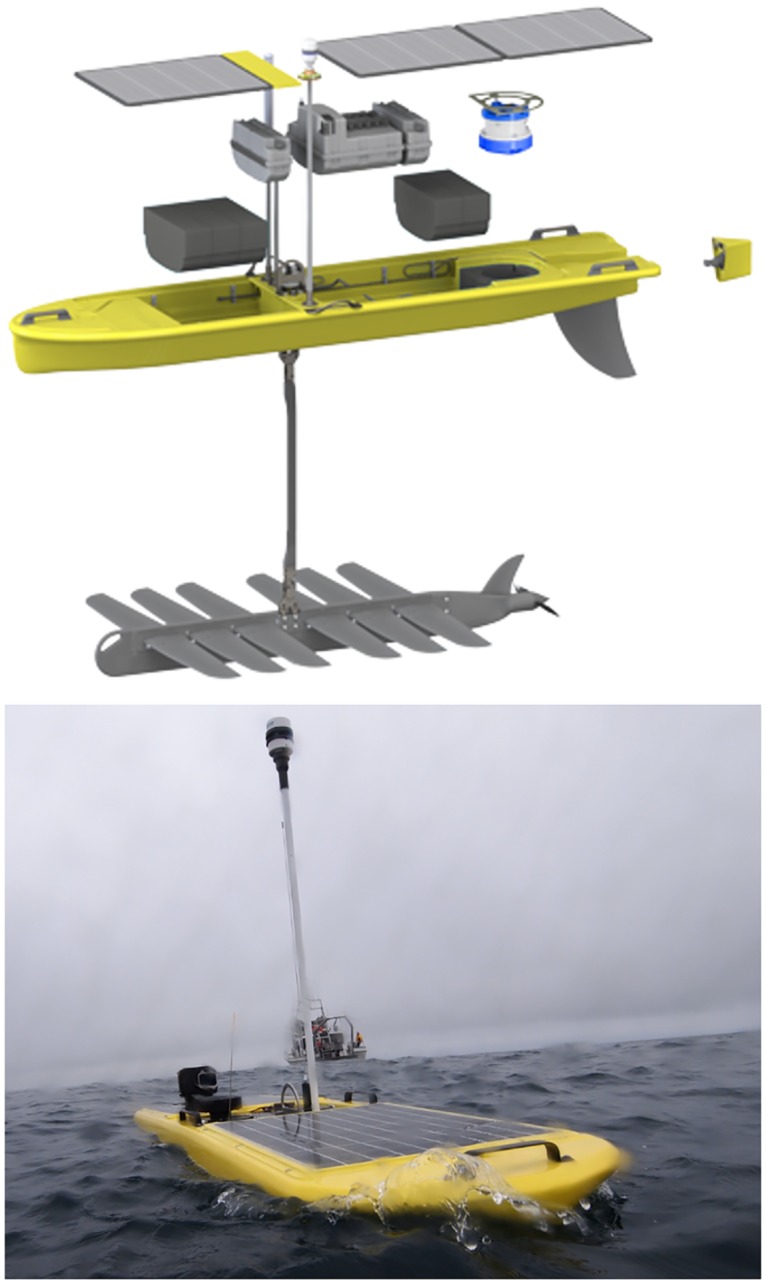
Wave Glider SV2. (Top) Blow-out showing the near-surface float housing the payload (including an ADCP (blue) and fluorometer), and connected to a sub (‘glider part’). The wave-induced friction between the two parts, connected through an umbilical, provides thrust. (Below) The Wave Glider in operation showing the antenna and solar panels. (Pictures courtesy of Liquid Robotics Inc.).

The Wave Glider is composed of two parts: a float which is roughly the size and shape of a surfboard and stays at the surface; and a sub having wings and hanging 4 m below the float on an umbilical tether (Fig 2). Because of the separation, the float experiences more wave motion than does the sub. This difference allows wave energy to be harvested to produce forward thrust (www.liquidrobotics.com). Iridium Satellite communication is used for command, control and data exfiltration, and GPS satellite transmissions for positioning. The USV was deployed and recovered with the oceanographic vessel RV Belgica, respectively on April 15^th^ and May 6^th^. Pilots controlled the Wave Glider from shore during the whole period (7 days a week, 24 hours a day).

#### Payload

Apart from navigation- and payload-control computers and satellite-communication systems, the Wave Glider was equipped with a fluorometer (Turner Designs, C3 submersible fluorometer), with sensors for measuring colour dissolved organic matter (CDOM) and crude and refined (poly- and mono-aromatic hydrocarbons) oil fluorescence, and for turbidity and water temperature just below the float of the Wave Glider. The fluorometer featured three optical sensors covering the spectrum from the deep ultraviolet to the infrared. The light-emitting diode for measuring turbidity from the scattering of light operated at a wavelength of 850 nm. Measured values were expressed in relative fluorescence units (RFU) (www.liquidrobotics.com).

Additionally, the float of the Wave Glider housed a broadband Acoustic Doppler Current Profiler (ADCP) (Teledyne/RD Instruments, 307.2 kHz). Current and acoustic backscatter data were acquired in three parts: part 1 with a vertical bin or cell size of 1 m, and glider motion removed, part 2 with a cell size of 2 m, because of an additional bottom track, and part 3 with similar settings as part 1. The Wave Glider had an average speed of 0.59 ms^-1^, with a maximum of 0.87 ms^-1^. ADCPs detect the echoes returned from suspended material (i.e. ‘sound scatterers’) from discrete depths of the water column. Echo intensities, per transmitted pulse, were recorded in counts (also termed the Received Signal Strength Indicator (RSSI), providing indirect information on the currents and density of suspended matter (‘backscatter’) within each ensonified bin.

#### Mission plan

The scientific goal of the mission was to characterise a shallow-water sandbank environment where intensive aggregate extraction takes place. On the one hand, background information was needed on the variability of natural advection and resuspension events. On the other hand, aggregate extraction is known to create dredge plumes; the challenge was to detect these plumes, as also their dispersal, and likely place of deposition. For this reason, the Wave Glider’s path was chosen to optimize the chance of characterizing both the natural and anthropogenic suspended sediment. In preparation of the mission, information was gathered on water depths, navigation hazards, vessel traffic, weather conditions and typical sea states. After accounting for technical exclusion zones (e.g., water depths shallower than -10 m, intensive shipping routes), a box was defined contouring the extraction site at a safety distance of at least 1 km (Fig 1). From a navigation-technical point of view, the Wave Glider was programmed with waypoints and headings to sail along the western (-37 m shallowest) and eastern (-39 m shallowest) lows during the ebbing (SW) and flooding (NE) phase of the tide, respectively. The southern (-16 m shallowest) and northern (-12 m shallowest) profiles crossed the sandbank. Pilots lengthened or shortened the Wave gliders’ path to sail those profiles under the most favourable tidal conditions, i.e., around slack water when currents were weakest, and never during spring ebb and flood. For these reasons, the Wave Glider undersampled the sandbank, providing little information on the hydrodynamics and sediment transport in the shallowest waters during high-energy conditions. The Wave Glider sailed for 22 days, completing 39 laps around the extraction site. Each lap took approximately 12.5 hours to complete, the length of the principal lunar semi-diurnal cycle. During this period, 28 extraction events took place.

### Data processing

#### Fluorescence data

The C3 turbidity RFU data were converted into Nepheloid Turbidity Units (NTU) after laboratory calibration (NTU = (RFU-6.9)/16.6) (pers. comm. Liquid Robotics Inc.). To obtain SPM mass concentration data in gl^-1^, NTU was further multiplied with a factor 1.6, which is a typical value derived from near-shore and offshore calibrations of optical turbidity sensors in Belgian waters [39].

#### Acoustic Doppler Current Profiler (ADCP)

For recalculation of bin depth to actual depth values, a draught of 0.25 m was applied for the distance of the ADCP below the water surface. The first bin that could be used was around -10 m only, because of contamination of the data in the upper water layers by the submerged part of the Wave Glider. Pulses were averaged into ensembles at a time interval of 60 seconds per sample. Together with an average platform speed of 0.59 ms^-1^, this resulted in an average horizontal resolution of 40 m.

The ADCP echo intensities, in dB, were corrected for beam spreading and water attenuation [40]. As with the ADCP current direction and magnitude data, the first bin started at -10 m water depth. To obtain rough estimates of mass concentration values, the dBs at -10 m were plotted against the C3 turbidity data (RFU). The assumption here is that the upper water column (first 10 m) has a uniform sediment concentration, so that the ADCP backscatter corresponds with the C3 turbidity data. For this conversion only RFU and dB data from calm periods (significant wave heights less than 1.4 m) were retained, and running-averages (20-min) were used in the linear regression analysis (resulting R² of 0.89). A second conversion, similar to that of the C3 data, was applied to transform the turbidity RFU into NTU (NTU = (RFU-6.9)/16.6) (pers. comm. Liquid Robotics Inc.), based on laboratory calibrations. The latter units were then also multiplied with a factor 1.6 to generate SPM concentration values in gl^-1^. The values were within an order of magnitude of those obtained from ship-borne measurements in the same period of the year and in the same area [41]. For further quantitative analyses, time series of currents and SPM were extracted at appropriate levels (e.g., representative for the upper and lower water layers, and depth-averaged). A running average was applied over a 20-min window.

### External data

#### MODIS Satellite data

The temporal variation of the C3 turbidity sensor, mounted in the Wave Glider float, was validated using imagery from the Moderate Resolution Imaging Spectroradiometer (MODIS) (via MUMM/GRIMAS extraction tool (http://www2.mumm.ac.be/remsem/timeseries/) [42]. The main motivation for this analysis was to have an independent dataset to verify and provide context for the variations in the field dataset. For each Wave Glider record, a nearest window of 25 pixels (1 km x 1 km) was defined. In the case of no clouds, an SPM concentration value was calculated at each of these pixels. For the correlation with the C3 data, a median MODIS-derived SPM value was retained when measurements were available for 13 of the 25 pixels, and when the measurement time difference between the Wave Glider and MODIS data was less than 2 hours. A median value was chosen to reduce bias from an ephemeral cloud cover and/or water glint. During the period of Wave Glider employment, the frequency of the daily image provision by MODIS was between 12h and 13h45.

#### Hydro-meteorological data

Wave information (e.g., significant wave height (H_s_ in m); and direction of low- and high-frequency waves (°), with a period less (H_z_) and more than 10 s, respectively) were obtained, at 30-min intervals, from a Wavec buoy (Flanders Hydrography) at 18 km southwest of the study area (MOW7 measuring pole; location see Fig 1). Wind climate, at 10-min intervals, was derived from the same pole. Water levels, current velocity and direction (10-min intervals) were extracted from an operational 3D hydrodynamic model [43]. On the basis of these data, the timing of high and low waters was extracted and transferred to the Wave Glider dataset.

#### Vessel monitoring data

To distinguish natural from human-induced variability in SPM concentration (e.g., caused by dredging, but also induced by wakes of nearby ships), ship-navigation data were analysed (Schelderadarketen, [44] and, where relevant, coupled to the time series (e.g., shortest distance to the Wave Glider). To detect dredging-induced sediment plumes, the timing of dredging activities was marked in the Wave Glider time series. During the Wave Glider experiment, 28 extractions took place using a trailer hopper dredger with a capacity of approximately 2500 m³. To enable discharge on the upper beach during the flood tide, all extractions were made during the ebbing phase of the tide.

All data were time-stamped to Universal Time Coordinates (UTC) allowing more easy correlation of various observations. Position coordinates were in UTM31 WGS84.

## Results

The Wave Glider’s time series provided a unique record of current and turbidity events over a period of 22 days. Analyses on current variability, and on external wave and wind data are summarised in the section on hydro-meteorological conditions. Next, turbidity events are described, first those that are thought to be naturally induced, and secondly those that could be related to the dredging activity. Quantification of hydro-meteorological conditions was particularly important in evaluating the sediment resuspension potential, and in constraining the magnitude and dispersal direction of the dredge plumes. In case of dominant SW dispersal of fines, the ecologically important gravel fields in the adjacent Habitat Directive area could be affected.

### Hydro-meteorological conditions

**Fig 3:**
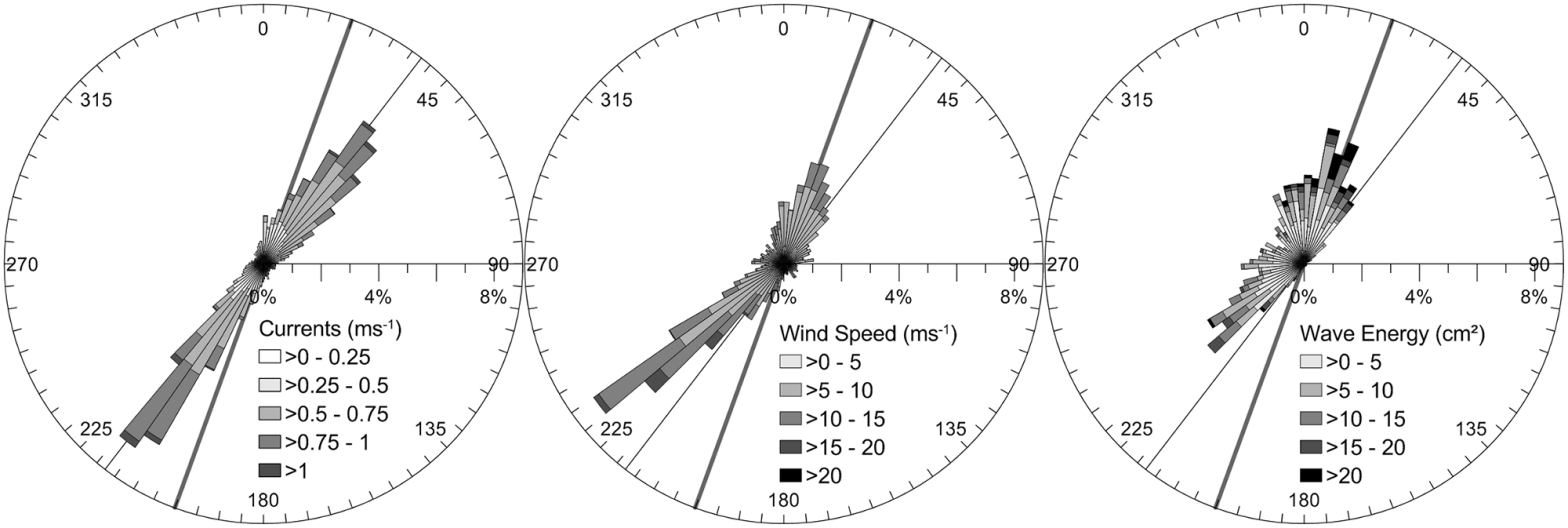
Hydro-meteorological conditions during the experiment. From left to right: Current velocity and direction (Wave Glider ADCP), wind velocity and direction, and low-frequency energy (frequency band of 0.03 H_z_ to 0.1 H_z_) and direction of low-frequency waves (H_z_ < 10 s) (MOW7 location, Fig 1). Bold line represents the axis of the sandbank; thin line is the axis of maximum currents.

During the Wave Glider experiment, hydro-meteorological conditions were rather calm, with waves exceeding 1 m only 28% of the time (H_s_ max = 2.60 m) (Fig 3). Mean tidal range increased from 3.67 m during neap to 4.73 m during spring conditions. Currents measured with the Wave Glider showed a 17° offset with the sandbank axis. The NE-directed flood and SW-directed ebb currents were more or less equal in strength, with the flood lasting somewhat longer (around 8%), and the ebb keeping its directionality for a longer period. Current velocities increased clearly from neap- to spring-tidal levels with surface values of up to 1.2 ms^-1^. In the deeper waters of the troughs, the surficial currents were approximately 21% stronger than those above the shallower sandbank slopes and crests. Winds blew mostly from a SW direction; winds of more than 20 ms^-1^ gave rise to high-frequency waves (H_z_ > 10 s) with an amplitude of more than 2 m (H_s_). This wave direction prevails in the BPNS. During employment, SW waves were present around 55% of the 22 days, whilst N to NE waves were active only 29% of the time. For low-frequency waves (H_z_ < 10 s), SW and NE conditions equalled. Significant wave heights were higher under SW conditions, though the low-frequency energy (0.03 H_z_ to 0.1 H_z_) of the northerly waves was significantly higher. This latter wave direction aligned with the sandbank’s axis (Fig 3).

### Natural variation of SPM concentration

#### Tidally-induced variation

**Fig 4:**
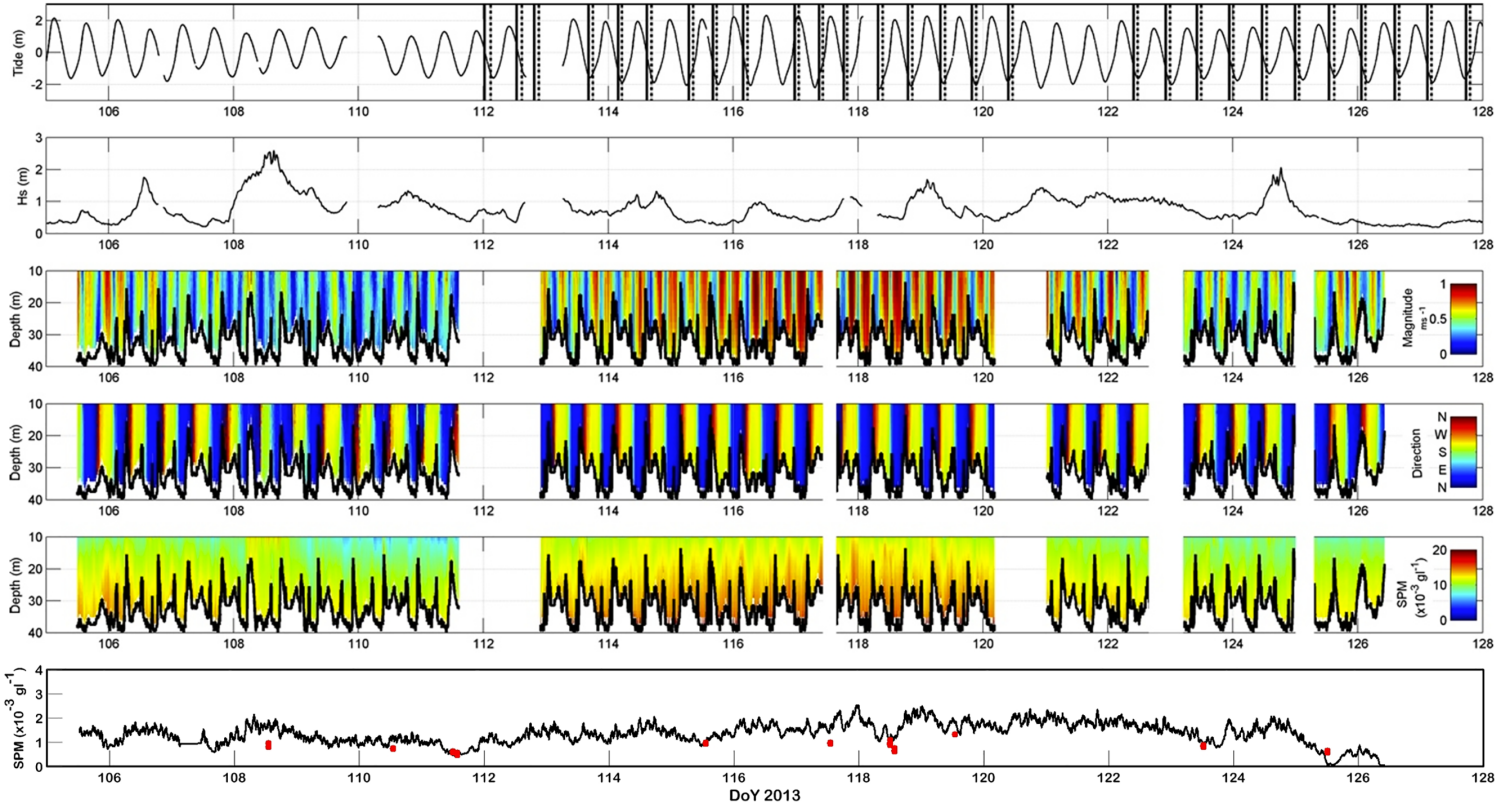
Composite of the Wave Glider measurements, together with the main hydro-meteorological conditions. From top to bottom, the figure shows: (1) water level, with the extraction events (28) superimposed; (2) significant wave height; (3) and (4) ADCP-derived current strength and direction; (5) ADCP-derived SPM concentrations; (6) surface SPM concentrations from the C3 sensor, superimposed with turbidity estimates derived from cloud-free MODIS satellite imagery data (red dots).

Peaks in SPM concentrations were linked mostly to peaks in current strength, both along the lows, parallel to the sandbank, and across its crest. Most obvious was the neap-to-spring variation (Fig 4). During spring tide, the ADCP-derived SPM concentrations were high throughout the water column, with highest values near the seabed. The time series of the surficial C3 fluorometer sensor, proxy for turbidity, showed a similar trend from neap to spring tide (Fig 4).

**Fig 5:**
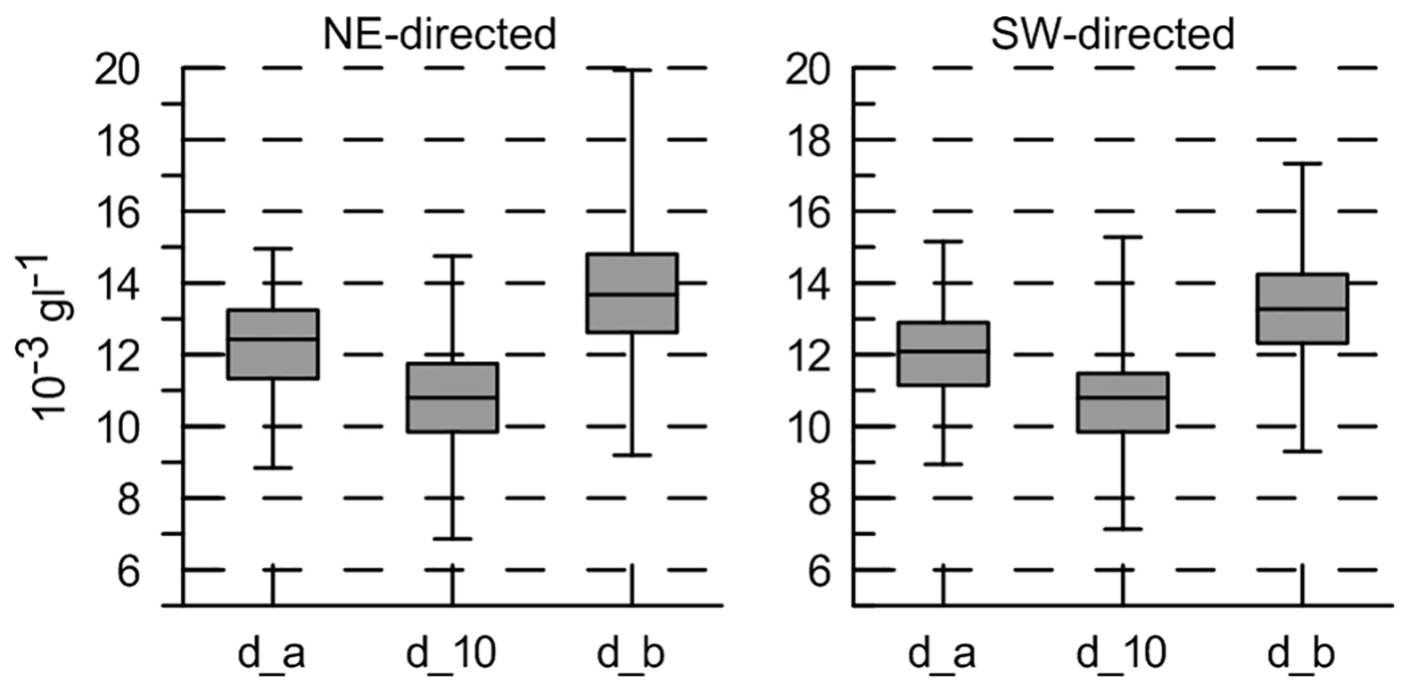
Boxplot of ADCP-derived SPM concentrations under NE- and SW-directed currents. Results are shown for currents stronger than 0.4 ms^-1^, being the sediment resuspension threshold. From left to right, values are depth-averaged (d_a); around -10 m (d_10); and near bottom (d_b). Values are most representative for SPM concentrations in the troughs fringing the sandbank.

SPM concentrations were similar under NE- and SW-directed currents (Fig 5), though slightly higher concentrations were measured under flood (NE) conditions. In the upper water layers, at -10 m, median values of SPM concentration reached about 0.010 gl^-1^; concentrations in the surface waters were around 0.001 to 0.002 gl^-1^ (Fig 4), for neap and spring tide respectively. SPM median concentrations in the lower waters were 0.011 to 0.015 gl^-1^ in the deepest areas and up to 0.019 gl^-1^ over the sandbank crests. However, peak concentrations were consistently missed, since the Wave Glider crossed the sandbanks under the most favourable conditions, with the weakest currents.

**Fig 6:**
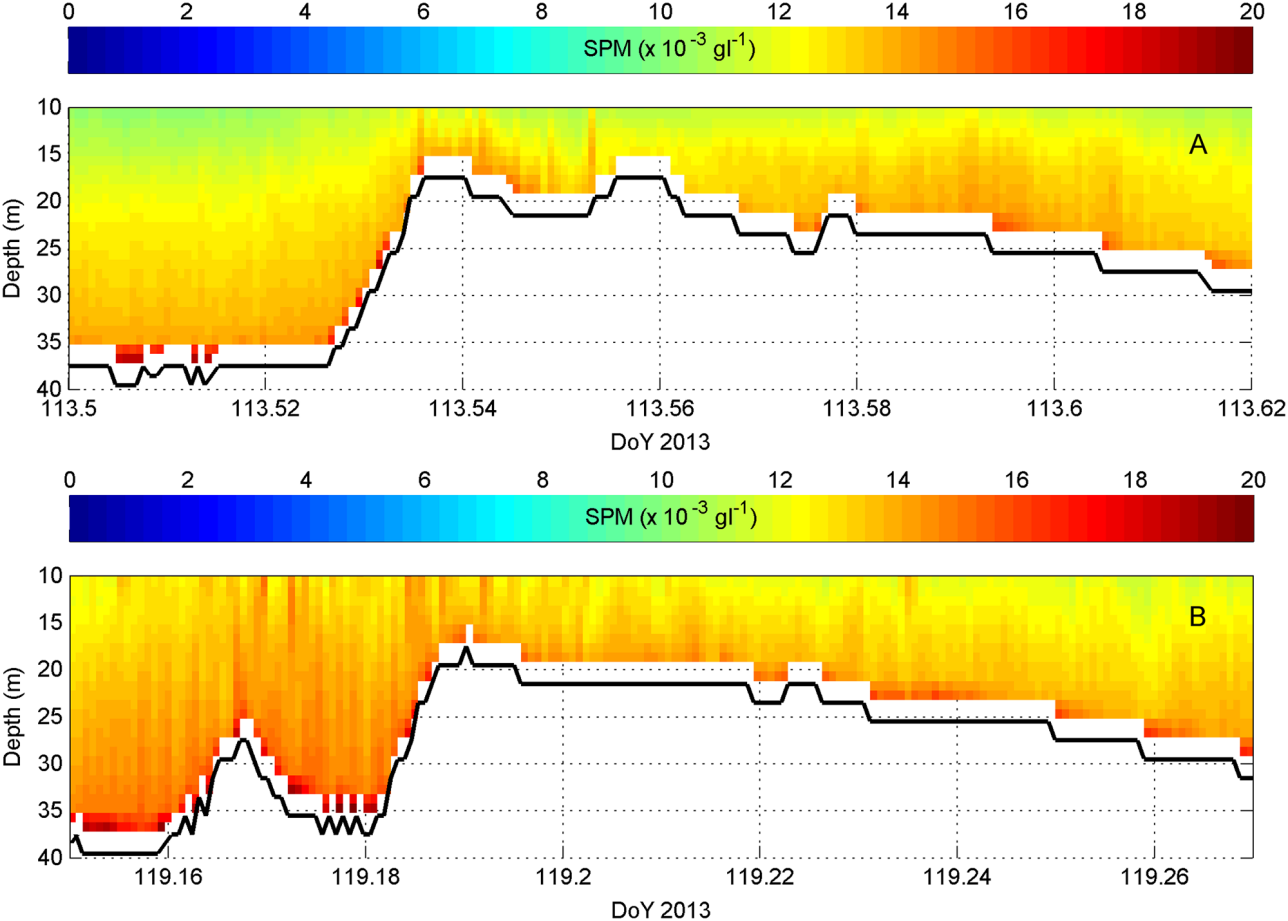
Examples of tide-topography interaction. (A): under low wave heights (Hs < 1 m; DoY 113.54)); (B): with wave interaction (Hs: 1–2 m; DoY 119.18). In both cases, the Wave Glider passed the top of the sandbank approximately 2.4 h after High Water. Location Oosthinder sandbank; northern cross-bank profiles in lower right inset of Fig 1.

At the few occasions that the sandbank was crossed at higher current velocities, tide-topography effects were observed resulting in resuspension (Fig 6), also in the lee sides of the superimposed bedforms.

#### Wave-induced variation

With increasing wave heights, higher SPM concentration values were derived, especially over topographic highs (Fig 6). Most obvious was a good correspondence between the values of the surface C3 turbidity sensor and the wave heights (Fig 4). Evaluating the whole time series of the C3, it was striking that, contrary to ADCP SPM values, the C3 values remained high after spring tide (DoY 120–123; Fig 4). During this period of 2.5 days wave heights were between 1 and 1.4 m, and originated consistently from the ENE direction. A mild storm occurred around DoY 108–109 (mid tide) (Fig 4). Waves originating from the SW reached a significant height of 2.6 m. The Wave Glider’s ADCP data showed a corresponding overall increase in current strengths, especially over the sandbank. Equally strong upper- and lower-water currents indicated strong mixing. SPM concentrations were raised not just over the sandbank, but also more regionally, suggesting that fine sediments advected away from the sandbank.

#### Human-induced variation

**Fig 7:**
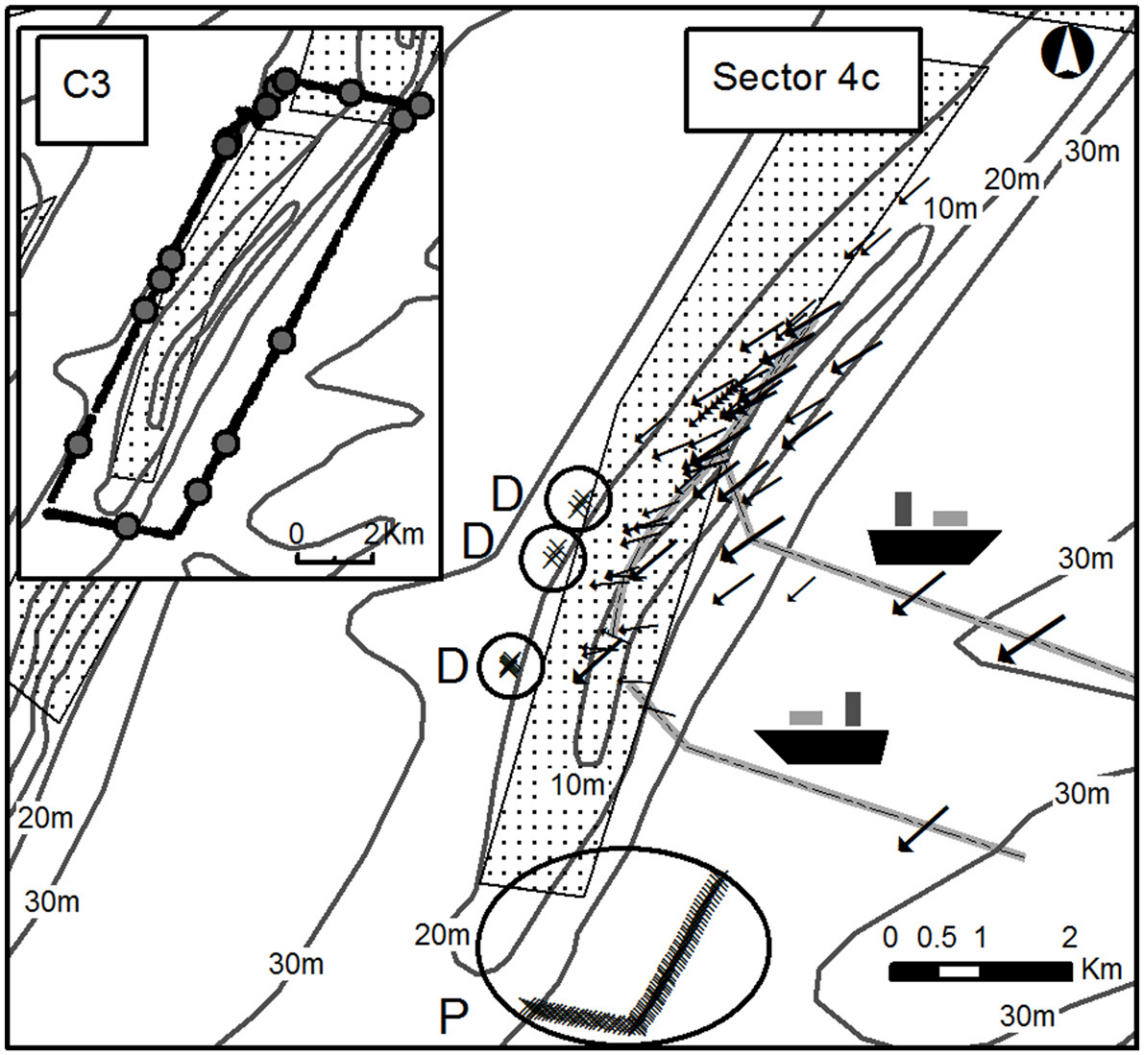
The concession area (dotted) with ADCP-derived locations of dredging-induced sediment plumes. Important SPM concentration anomalies were observed along the western edge of the sandbank: in the circles ‘D’, these suggest the occurrence of dynamic plumes (x); in circle ‘P’, a passive plume (x). Modelled surface current vectors (arrows; 10-min averaged) during the extraction events were all SW-directed. One typical aggregate extraction pathway is shown in grey. In the inset, C3-derived surface turbidity values are shown for the tour in which the ADCP detected the passive plume (circle P). The largest dots represent higher SPM concentration amounts. Note, that no consistently high surface concentrations were recorded, pointing to a mid-water position of the passive plume. For location of the C3 inset, note the position of the data in respect to the delineation of Sector 4c (dotted) in the main figure.

**Fig 8:**
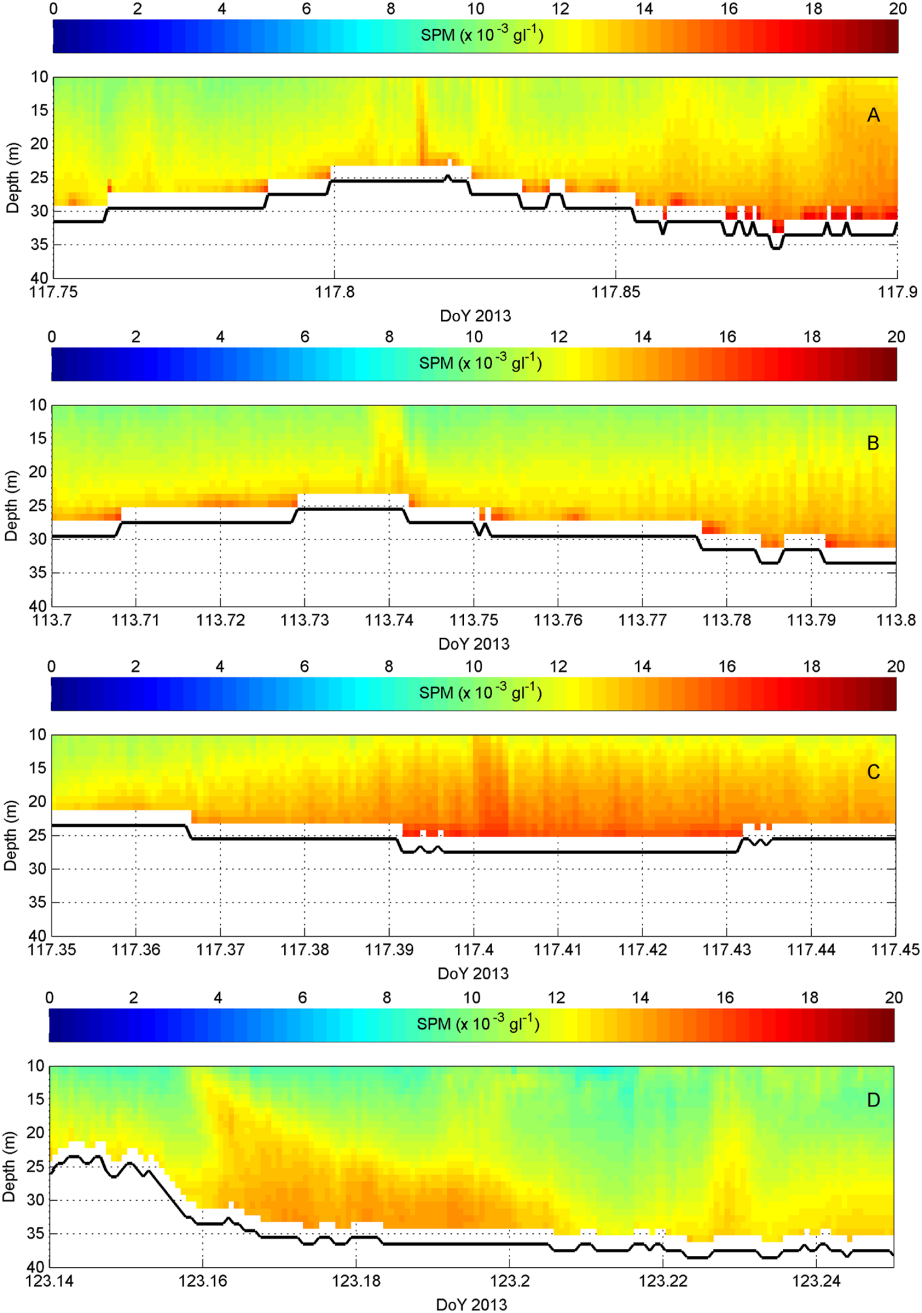
Examples of dredging-induced sediment plumes (from ADCP backscatter data) (A-B-C-D). Locations are from North to South in Fig 7, and correspond to (1) Dynamic plumes ‘D’ (DoY 117.815 (A), 113.74 (B), 117.4 (C)), as observed close to the dredging vessel (< 600 m). Dimension of the plumes was less than 140 m; and (2) Passive plume ‘P’ (DoY 123.16 (D)), observed in the far field, and transported by the SW-directed ebb tidal current. Note that DoY 123.14–123.177 (minimum 780 m wide) was cross-sandbank oriented; afterwards the Wave Glider sailed parallel to the sandbank, for approximately 1.8 km (see Fig 7). Relating to this passive plume, the last extraction event was 7.8 km away from the Wave Glider.

During the Wave Glider monitoring period, 28 extractions were made using a small dredging vessel of approximately 2500 m^3^. Generally, the Wave Glider was 0.5 to 1 km away from the vessel. This distance was too far to detect larger scale differences in SPM concentrations before, during and after the dredging. Important anomalies in SPM concentration values did suggest the detection of individual dredging-induced surface, dynamic and passive plumes, and unambiguously showed the descent of such plumes from the upper waters to the seabed. Fig 7 shows where these anomalies were depicted, whilst Fig 8 visualizes them. SPM concentrations in the surface plumes, containing released fines, were difficult to quantify, due to dispersal and to uncertainty in the nature of the increases compared to other influences, such as air bubbles. However, dynamic plumes were visualised clearly when the Wave Glider was close to the dredging vessel (i.e., less than 600 m away) and when currents were directed towards the Wave Glider. These dynamic plumes suggest deposition of the main overflow from the dredging vessel. Increases in SPM concentrations were measured over a distance of around 120 m and were a factor of 1.25 greater than the natural background values. Most intriguingly, a passive plume was observed also, around 3 hours after the preceding extraction event, 7.8 km away. The position of this plume corresponded well with modelled predictions of deposition that took into account the measured current velocities and directions in the area. During and after the dredging event, currents (around 0.7 ms^−1^) were directed to the SW and were reinforced by the waves; winds blew in the same direction. No other ships were nearby.

## Discussion

### Wave Glider as monitoring platform

Overall, the Wave Glider proved to be a stable platform for monitoring both naturally and human-induced variability in hydrodynamic and sediment processes. Natural resuspension and advection were successfully observed under tidally and wave-induced currents. Most importantly, the instruments on the Wave Glider allowed identification of well-delineated sediment plumes resulting from marine aggregate extraction. Advantages as well as disadvantages are summarised in more detail in Table 1.

**Table 1:** Pros and cons determined from our Wave Glider monitoring experiment.

**Pros**
Stable platform in a tidally and wave-influenced energetic environment
Simultaneous operation of *ad hoc* sensors for an integrated spatio-temporal dataset
Long-term quasi continuous data series, covering natural variability and human-induced effects
High-frequency measurements for high spatial resolution
Opportunity for event detection, by measuring effects from any phase of the event, including lag effects
Effective remote control by pilots, avoiding collisions in busy traffic and optimising the Wave Glider’s performance by taking account of the tides
Cost-effectiveness
**Cons**
Limitations related to survey design: Unequal representation of conditions, with more data obtained in the troughs than over the sandbank ridges, which were crossed around slack water, missing out on the highest-turbidity events
Low temporal resolution per sublocation, because of the lap time of the trajectory compared to the tidal oscillation
Detection of events, but no quantification of their dilution rate
Lack of calibration and validation, critical for quality assurance
Additional datasets needed for balanced evaluations of environmental conditions
Need for continuous piloting

The most important strengths and added value of the Wave Glider were its endurance and its versatile platform, allowing for the integrated operation of *ad hoc* sensors. The combined use of a C3 optical sensor for surficial turbidity and an ADCP that acoustically measured turbidity throughout the water column showed high promise, and enabled comparisons with satellite-derived turbidity data. However, as in earlier studies in the area [45,46]), results showed that the surface-turbidity values were not reliable indicators of sediment advection and resuspension in the water column as shown by the ADCP (Fig 4). This limitation is an important consideration when using satellite data for the monitoring of turbidity [47].

Data quality (i.e., internal consistency) was overall very good, but deteriorated under higher wave events. Values of the surface C3 sensor followed a similar trend as the wave height. Although, it is plausible that more sediments are advected away from the sandbanks under highly than under lowly energetic wave conditions [48,49,50], it cannot be excluded that wave-induced pitch-and-roll movements led to trapping of air bubbles on the optical face of the sensor, and therefore to overestimations of sediment load. Next-generation fluorometers (www.turnerdesigns.com) with air purge slots in shade caps should be used in future surveys. The ADCP data may also have been biased by air bubbles [51]. At higher wave heights (mostly > 2 m), ADCP data increasingly showed bands of anomalous backscatter values in the upper part of the water column, though values normalised farther down the water column.

Most of the disadvantages listed in Table 1 were inherent to the survey design, and difficult to account for in the analyses. Since nearby extractions were on-going during the experiment, the Wave Glider had to sail in rectangular laps around the extraction sector on the sandbank, resulting in bank-parallel sections of around 12 km in the troughs, separated by much shorter cross-bank transects of around 3 km. It needs reiteration that the Wave Glider sailed with the tides: the western part of the lap was always sailed during ebb, and the eastern part always during flood. Since the sandbank was crossed mostly during slack water, important peak SPM concentrations over the sandbanks were missed; hence, the final dataset is marked by an unequal representation of different-strength forces acting on the sandbank area.

The spatial extent of the aggregate-extraction sector was such that one lap by the Wave Glider took 10–15 hrs, meaning that each location was sampled only once during a tidal cycle (12.25 hrs) on average. The measured changes in sediment concentrations were biased by the spatial position of the Wave Glider with the tidal phase. For a regional characterisation of natural background conditions, such limited temporal resolution is acceptable. If sandbank dynamics or detailed impacts need quantification, however, short laps or transects and higher sampling frequencies for each location are favourable.

The Wave Glider captured a few dynamic plumes when it was close enough to a dredging vessel in operation. However, when quantification of the behaviour (e.g., particle size and nature), dispersal and dilution of sediment plumes is targeted, monitoring from ships is more informative because these can be equipped with a more complete set of instruments and more easily manoeuvred to stay within a plume [31,33,34,35,52]. Uniquely, the Wave Glider’s ADCP detected the descent of a passive plume. Such plumes combine fines released from the surface as well as from dynamic plumes, and travel in the middle part of the water column [31]. A passive plume was detected only once over the entire time series during which 28 extraction events (day and night) took place. Owing to the large space and time lag with respect to the time and location of the corresponding dredging activities, this plume would most probably have been difficult to detect using a ship with typically shorter monitoring periods. Even a Wave Glider, which can be operational for much longer monitoring periods, will only depict human-induced SPM increases when the platform crosses the sediment plume, which lies downstream of its originating dredging event. The migration and dispersal of plumes are governed by highly variable hydro-meteorological conditions and therefore difficult to predict. Measurements at fixed locations (e.g., with multi-sensor benthic landers) also provide little chance to detect dredging-induced sediment plumes, despite providing long time series at very high temporal resolution. In trying to capture and understand sediment plumes, Wave Gliders can play an important role, but there will always be a trade-off between the desired temporal versus spatial resolution and simple versus a more complete set of instrumentation. If possible, a flexible, long-term monitoring strategy is followed, taking full advantage of the complementarity of autonomous vehicles, landers and ship-based observations.

At least for the time being, ships remain important for *in situ* calibration of all sensor data (e.g., water sampling and other instrumentation to determine nature, size and concentration of SPM) and for more complete synchronous measurements (e.g., multibeam bathymetry and backscatter) than are currently possible with Wave Gliders. However, with increasing use of autonomous platforms, development and optimisation of sensors and other equipment suitable for use on Wave Gliders are on-going. Promising examples are the incorporation of small-sized water samplers for calibration of sensor data [53], and experiments in towing light-weight hydro-acoustic instruments for high-resolution depth and sonar registrations [54].

### Time-series analyses

Wave Glider monitoring generates time series from multiple instruments that offer new possibilities for process-response analysis. In our study, important SPM events could be visualised, especially when the colour scale of images was fine-tuned to show the highest contrast within the range of values that mattered most. It proved difficult, however, to find significant quantitative correlations between the long time series and the main processes driving SPM concentrations and transport. Overall patterns were obvious, but quantitative links were mostly biased by interference of multiple processes, including noise.

An important source of bias is the overestimation of ADCP-derived SPM-concentration values. Variations in echo-intensity data are not an exclusive function of suspended sediments, but relate to a mixture of sources with individual contributions that are hard to disentangle [55,56]. Correlations with tides, currents and waves, which are easiest to hindcast, are overprinted by much more unpredictable or even random effects of debris, phyto- and zooplankton [57,58], mammals, air bubbles [51] and noise of ships [59]. Ideally, every effort should be made to constrain the uncertainties associated with these effects. In our study, all SPM events that were interpreted as dredge plumes were verified first against current- and wave influence and against ship passages. The Wave Glider’s pitch-and-roll information was used additionally, to evaluate the chance of encountering spikes. Biological influence was also observed in the dataset, be it indirectly. Around the timing of the phytoplankton bloom, as reported around DoY 123.5 (Fig 4) from MODIS-derived Chlorophyll-a values, our ADCP SPM data showed wisps of high backscatter in the upper water layers (up to a water depth of -20 m; 10 m above seabed). Other possible causes for this shallow backscatter anomaly were excluded: wave heights were around 0.5 m only, and the nearest ships were more than 4 km away; values for colour dissolved organic matter, as measured by the Wave Glider, and a useful proxy for debris from dead organic matter [60] were low too.

To discriminate human forcing in the time series, it is important to note that extraction-induced dynamic sediment plumes may have a limited spatial extent, no more than 2*60-second ensembles in our Wave Glider SPM data, and are ephemeral in nature. During the experiment, the human-induced increases in SPM values fell within their natural range (for a relative small dredging vessel of 2500 m^3^). Thus, these events are missed easily in autonomously (USV, AUV) recorded time series, especially if automated routines would be used for their identification. Due to its much larger dimension, the detection of the passive plume was straightforward. For such plumes, though, correlation with a source and with processes governing its advection is complicated by the large space and time lag with respect to the preceding dredging event.

Hydro-meteorological forcing drives the dispersal of all plumes [31] and needs to be accounted for when evaluating plume events. In the present case, extraction occurred consistently during ebb, limiting the transport of plumes to SW directions. Using this knowledge, some SPM events, as measured by the Wave Glider, could not be due to the dredging.

The identification of the overall SW-directed transport of the sediment plumes was very important as it showed that the probability of deposition of fines in the Habitat Directive area, only 2.5 km southwards of the extraction site, was high. The potential impact of these fines on the ecologically valuable gravel habitats is now under investigation. In this light, future plume research will focus on more advanced modelling of their spatial dimensions, dispersal pathways and depositional patterns. The Wave Glider data series, supplemented by other sensor observations and by ground-truthing, will be pivotal in the validation of these plume-dispersal models. The present dataset was already used to select and sample seabed areas where human-induced changes in habitat characteristics would be most likely, given the on-going extraction activities. Should consistent deposition patterns be found, fining of surface sediments or even smothering of habitats is of considerable concern. Any significant net deposition within the downdrift Habitat Directive area, hosting sensitive and unique habitats, will necessitate adaptation of the dredging practices (e.g., alternating between extraction locations or no persistent dredging during ebb).

## Conclusions

Through careful planning and 24-hr piloting, a Wave Glider was employed successfully in one of Worlds’ most heavily navigated and exploited sea areas, recording long time series of natural and human-induced spatio-temporal variability in various parameters. Using the Wave Glider data, it was possible to identify and evaluate human-induced sediment plumes in light of natural, tidally and wave-induced forcing on SPM concentrations and on sediment-transport directions.

During the Wave Glider experiment, 28 extractions took place. The effect of only a few was observed in the dataset, mostly due to an overall distance of 0.5 to 1 km to the dredging vessel. After careful evaluation of all potential sources, instruments did depict dredging-induced increases in turbidity, but overall the concentrations fell within the limits of natural variation. Dimensions of well-delineated dredging-induced dynamic and passive sediment plumes were assessed, as also was their deposition area. In the near field, quasi-immediate deposition is suggested of the main overflow, whilst finer-grained material, segregated from the main plume and the erosion around the draghead, ended up in the far field. The latter formed the passive plume, resided temporarily below the middle of the water column, and was deposited three hours after the last extraction activity. The spatio-temporal pattern of far-field spreading was in agreement with the prevailing hydro-meteorological forcing.

For the monitoring of sediment processes and dredge plumes, a flexible monitoring strategy is recommended that combines short- and long-term measurements from mobile platforms and at fixed locations in carefully considered survey designs. Such an approach ensures that predictable events and processes are quantified, including spatio-temporal background conditions and the dilution of observed SPM increases. Long-term measurements are needed to increase the likelihood that unpredictable or random events and processes are captured. From a time and cost perspective, the Wave Glider proved valuable in environmental monitoring of sediment processes, and aided in the optimisation of follow-on monitoring and research of processes of which the knowledge base is still too fragmented. For the time being, ship-borne measurements remain essential for calibration and validation of the sensor data, but on-going technological developments in Wave Glider construction and instrumentation will increase the stand-alone value of its measurements.

## References

[1] Borja A, Bricker SB, Dauer DM, Demetriades NT, Ferreira JG, Forbes AT, et al Overview of integrative tools and methods in assessing ecological integrity in estuarine and coastal systems worldwide. Marine Pollution Bulletin. 2008;56: 1519–1537. 10.1016/j.marpolbul.2008.07.005 18715596

[2] Morris D, O’Brien C, Larcombe P. Actually achieving marine sustainability demands a radical re-think in approach, not “more of the same”. Marine Pollution Bulletin. 2011;62: 1053–1057. 10.1016/j.marpolbul.2011.02.027 21397916

[3] Borja Á, Elliott M, Carstensen J, Heiskanen A-S, van de Bund W. Marine management—Towards an integrated implementation of the European Marine Strategy Framework and the Water Framework Directives. Marine Pollution Bulletin. 2010;60: 2175–2186. 10.1016/j.marpolbul.2010.09.026 20965524

[4] Tallis H, Levin PS, Ruckelshaus M, Lester SE, McLeod KL, Fluharty DL, et al The many faces of ecosystem-based management: Making the process work today in real places. Marine Policy. 2010;34: 340–348. 10.1016/j.marpol.2009.08.003

[5] Rice J, Arvanitidis C, Borja A, Frid C, Hiddink JG, Krause J, et al Indicators for Sea-floor Integrity under the European Marine Strategy Framework Directive. Ecological Indicators. 2012;12: 174–184. 10.1016/j.ecolind.2011.03.021

[6] De Jonge VN, Elliott M, Brauer VS. Marine monitoring: Its shortcomings and mismatch with the EU Water Framework Directive’s objectives. Marine Pollution Bulletin. 2006;53: 5–19. 10.1016/j.marpolbul.2005.11.026 16426645

[7] Binot J, Daňobeita J, Muller T, Nieuwejaar PW, Rietveld MJ, Stone P. European Ocean Research Fleets—Towards a Common Strategy and Enhanced Use Strasbourg, France: Marine Board-ESF; 2007 p. 62 Marine Board Position Paper 10.

[8] Petersen W. FerryBox systems: State-of-the-art in Europe and future development. Journal of Marine Systems. 2014;140, Part A: 4–12. 10.1016/j.jmarsys.2014.07.003

[9] Degrendele K, Roche M, Schotte P, Lancker VRMV, Bellec VK, Bonne WMI. Morphological Evolution of the Kwinte Bank Central Depression Before and After the Cessation of Aggregate Extraction. Journal of Coastal Research. 2010; 77–86.

[10] Van Lancker V, Moerkerke G, Du Four I, Verfaillie E, Rabaut M, Degraer S. 14—Fine-Scale Geomorphological Mapping of Sandbank Environments for the Prediction of Macrobenthic Occurrences, Belgian Part of the North Sea In: Baker PTHK, editor. Seafloor Geomorphology as Benthic Habitat. London: Elsevier; 2012 pp. 251–260. Available: http://www.sciencedirect.com/science/article/pii/B9780123851406000141

[11] Brown CJ, Blondel P. Developments in the application of multibeam sonar backscatter for seafloor habitat mapping. Applied Acoustics. 2009;70: 1242–1247. 10.1016/j.apacoust.2008.08.004

[12] Harris PT, Baker EK, editors. GEOHAB Atlas of Seafloor Geomorphic Features and Benthic Habitats [Internet]. London: Elsevier; 2012 Available: http://www.sciencedirect.com/science/article/pii/B9780123851406000670

[13] Cacchione DA, Sternberg RW, Ogston AS. Bottom instrumented tripods: History, applications, and impacts. Continental Shelf Research. 2006;26: 2319–2334. 10.1016/j.csr.2006.07.027

[14] Palinkas CM, Ogston AS, Nittrouer CA. Observations of event-scale sedimentary dynamics with an instrumented bottom-boundary-layer tripod. Marine Geology. 2010;274: 151–164. 10.1016/j.margeo.2010.03.012

[15] Sternberg RW, Aagaard K, Cacchione D, Wheatcroft RA, Beach RA, Roach AT, et al Long-term near-bed observations of velocity and hydrographic properties in the northwest Barents Sea with implications for sediment transport. Continental Shelf Research. 2001;21: 509–529. 10.1016/S0278-4343(00)00103-5

[16] Black KS, Fones GR, Peppe OC, Kennedy HA, Bentaleb I. An autonomous benthic lander:: preliminary observations from the UK BENBO thematic programme. Continental Shelf Research. 2001;21: 859–877. 10.1016/S0278-4343(00)00116-3

[17] Favali P, Beranzoli L. Seafloor Observatory Science: a Review. Ann Geophys. 2006;49 10.4401/ag-3125

[18] Goff JA, Mayer LA, Traykovski P, Buynevich I, Wilkens R, Raymond R, et al Detailed investigation of sorted bedforms, or “rippled scour depressions,” within the Martha’s Vineyard Coastal Observatory, Massachusetts. Continental Shelf Research. 2005;25: 461–484. 10.1016/j.csr.2004.09.019

[19] Monna S, Falcone G, Beranzoli L, Chierici F, Cianchini G, De Caro M, et al Underwater geophysical monitoring for European Multidisciplinary Seafloor and water column Observatories. Journal of Marine Systems. 2014;130: 12–30. 10.1016/j.jmarsys.2013.09.010

[20] Sternberg RW, Nowell ARM. Continental shelf sedimentology: scales of investigation define future research opportunities. Journal of Sea Research. 1999;41: 55–71. 10.1016/S1385-1101(98)00037-9

[21] Manley JE. Unmanned surface vehicles, 15 years of development OCEANS 2008. IEEE; 2008 pp. 1–4. Available: http://ieeexplore.ieee.org/xpls/abs_all.jsp?arnumber=5152052

[22] Villareal TA, Wilson C. A Comparison of the Pac-X Trans-Pacific Wave Glider Data and Satellite Data (MODIS, Aquarius, TRMM and VIIRS). PLoS ONE. 2014;9: e92280 10.1371/journal.pone.0092280 24658053 PMC3962394

[23] Hull, T., Sivyer, D. Wave Glider trial, final report. September 2013 [Internet]. 2013 p. 18. Available: http://www.cefas.defra.gov.uk/publications/files/WaveGliderReport_Cefas_11Sept2013.pdf

[24] Miles T, Glenn SM, Schofield O. Temporal and spatial variability in fall storm induced sediment resuspension on the Mid-Atlantic Bight. Continental Shelf Research. 2013;63, Supplement: S36–S49. 10.1016/j.csr.2012.08.006

[25] Piterbarg L, Taillandier V, Griffa A. Investigating frontal variability from repeated glider transects in the Ligurian Current (North West Mediterranean Sea). Journal of Marine Systems. 2014;129: 381–395. 10.1016/j.jmarsys.2013.08.003

[26] Fong DA, Jones NL. Evaluation of AUV-based ADCP measurements. Limnology and Oceanography: Methods. 2006;4: 58–67.

[27] Foster SD, Hosack GR, Hill NA, Barrett NS, Lucieer VL. Choosing between strategies for designing surveys: autonomous underwater vehicles. Methods Ecol Evol. 2014;5: 287–297. 10.1111/2041-210X.12156

[28] Wynn RB, Huvenne VAI, Le Bas TP, Murton BJ, Connelly DP, Bett BJ, et al Autonomous Underwater Vehicles (AUVs): Their past, present and future contributions to the advancement of marine geoscience. Marine Geology. 2014;352: 451–468. 10.1016/j.margeo.2014.03.012

[29] Daniel T, Manley J, Trenaman N. The Wave Glider: enabling a new approach to persistent ocean observation and research. Ocean Dynamics. 2011;61: 1509–1520. 10.1007/s10236-011-0408-5

[30] Van Lancker V, Bonne WMI, Garel E, Degrendele K, Roche M, DV den Eynde, et al Recommendations for the sustainable exploitation of tidal sandbanks. Journal of Coastal Research. 2010; 151–164.

[31] Spearman JR, De Heer A, Aarninkhof SGJ, Van Koningsveld M. Validation of the TASS system for predicting the environmental effects of trailing suction hopper dredgers. Terra et Aqua. 2011;125: 14–22.

[32] Newell RC, Hitchcock DR, Seiderer LJ. Organic Enrichment Associated with Outwash from Marine Aggregates Dredging: A Probable Explanation for Surface Sheens and Enhanced Benthic Production in the Vicinity of Dredging Operations. Marine Pollution Bulletin. 1999;38: 809–818. 10.1016/S0025-326X(99)00045-4

[33] Hitchcock DR, Bell S. Physical Impacts of Marine Aggregate Dredging on Seabed Resources in Coastal Deposits. Journal of Coastal Research. 2004; 101–114. 10.2112/1551-5036(2004)20[101:PIOMAD]2.0.CO;2

[34] Smith SJ, Friedrichs CT. Size and settling velocities of cohesive flocs and suspended sediment aggregates in a trailing suction hopper dredge plume. Continental Shelf Research. 2011;31: S50–S63. 10.1016/j.csr.2010.04.002

[35] Duclos P-A, Lafite R, Le Bot S, Rivoalen E, Cuvilliez A. Dynamics of Turbid Plumes Generated by Marine Aggregate Dredging: An Example of a Macrotidal Environment (the Bay of Seine, France). Journal of Coastal Research. 2013; 25–37. 10.2112/JCOASTRES-D-12-00148.1

[36] Van Lancker V, Baeye M, Dimitris Evangelinos, Van den Eynde D. Monitoring of the impact of the extraction of marine aggregates, in casu sand, in the zone of the Hinder Banks Scientific Report 2—January—December 2014. Brussels: Royal Belgian Institute of Natural Sciences, OD Nature; 2015 p. 74 pp. +5 Annexes.

[37] Haelters J, Kerckhof F, Houziaux JS. The designation of marine protected areas in the Belgian part of the North Sea: a possible implementation of OSPAR Recommendation 2003/3 in Belgium. Brussels: Royal Belgian Institute of Natural Sciences, OD Nature; 2007 p. 46.

[38] Liquid Robotics, Inc. Wave Glider (Model 08) User Manual. Version 2.41. 2010 p. 190.

[39] Fettweis M. Uncertainty of excess density and settling velocity of mud flocs derived from in situ measurements. Estuarine, Coastal and Shelf Science. 2008;78: 426–436. 10.1016/j.ecss.2008.01.007

[40] Deines KL. Backscatter estimation using Broadband acoustic Doppler current profilers. Proceedings of the IEEE Sixth Working Conference on Current Measurement, 1999. 1999 pp. 249–253. 10.1109/CCM.1999.755249

[41] Van Lancker V, Baeye M, Fettweis M, Francken F, Van den Eynde D. Monitoring of the impact of the extraction of marine aggregates, in casu sand, in the zone of the Hinder Banks. Brussels: Royal Belgian Institute of Natural Sciences, OD Nature; 2014 p. 46 pp. + 9 Annexes.

[42] Vanhellemont Q, Nechad B, Ruddick K. GRIMAS: gridding and archiving of satellite-derived ocean colour data for any region on earth. Proceedings of the CoastGIS 2011 conference held in Ostend. 2011 pp. 5–8.

[43] Luyten PJ, Jones JE, Proctor R, MUMM. A coupled hydrodynamical-ecological model for regional and shelf seas: User Documentation. Brussels: Royal Belgian Institute of Natural Sciences, OD Nature; 2011 p. 1177.

[44] Van den Branden R, De Schepper G, Naudts L. Automatische registreersystemen geïnstalleerd aan boord van de zandwinningsschepen: overzicht van de verwerkte data van het jaar 2012. Brussels: Royal Belgian Institute of Natural Sciences, OD Nature; 2013.

[45] Fettweis M, Nechad B, Van den Eynde D. An estimate of the suspended particulate matter (SPM) transport in the southern North Sea using SeaWiFS images, in situ measurements and numerical model results. Continental Shelf Research. 2007;27: 1568–1583. 10.1016/j.csr.2007.01.017

[46] Fettweis MP, Nechad B. Evaluation of in situ and remote sensing sampling methods for SPM concentrations, Belgian continental shelf (southern North Sea). Ocean Dynamics. 2011;61: 157–171. 10.1007/s10236-010-0310-6

[47] Chen Z, Hu C, Muller-Karger F. Monitoring turbidity in Tampa Bay using MODIS/Aqua 250-m imagery. Remote Sensing of Environment. 2007;109: 207–220. 10.1016/j.rse.2006.12.019

[48] Williams J.J., Humphery JD, Hardcastle PJ, Wilson DJ. Field observations of hydrodynamic conditions and suspended particulate matter in the southern North Sea. Continental Shelf Research. 1998;18: 1215–1233. 10.1016/S0278-4343(98)00041-7

[49] Vincent CE, Stolk A, Porter CFC. Sand suspension and transport on the Middelkerke Bank (southern North Sea) by storms and tidal currents. Marine Geology. 1998;150: 113–129. 10.1016/S0025-3227(98)00048-6

[50] Giardino A, Van den Eynde D, Monbaliu J. Wave effects on the morphodynamic evolution of an offshore sand bank. Journal of Coastal Research. 2010;51: 127–140.

[51] Guerrero M, Rüther N, Szupiany RN. Laboratory validation of acoustic Doppler current profiler (ADCP) techniques for suspended sediment investigations. Flow Measurement and Instrumentation. 2012;23: 40–48. 10.1016/j.flowmeasinst.2011.10.003

[52] Wood JD, Boye D. Monitoring Suspended Sediment Plumes Using an Acoustic Doppler Current Profiler. OCEANS 2007. 2007 pp. 1–7. 10.1109/OCEANS.2007.4449165

[53] Ferri G, Manzi A, Fornai F, Ciuchi F, Laschi C. The HydroNet ASV, a Small-Sized Autonomous Catamaran for Real-Time Monitoring of Water Quality: From Design to Missions at Sea. IEEE Journal of Oceanic Engineering. 2014;PP: 1–17. 10.1109/JOE.2014.2359361

[54] Munday E, Acker T, Dawson J. Specialized Tools for Biological Assessment Using Split Beam Hydroacoustics. 144th Annual Meeting of the American Fisheries Society. Afs; 2014. Available: ftp://dns.soest.hawaii.edu/bhowe/outgoing/IEEEOES_2013/papers/130503-136.pdf

[55] Thorne PD, Hanes DM. A review of acoustic measurement of small-scale sediment processes. Continental Shelf Research. 2002;22: 603–632. 10.1016/S0278-4343(01)00101-7

[56] Gartner JW. Estimating suspended solids concentrations from backscatter intensity measured by acoustic Doppler current profiler in San Francisco Bay, California. Marine Geology. 2004;211: 169–187. 10.1016/j.margeo.2004.07.001

[57] Lorke A, McGinnis DF, Spaak P, Wüest A. Acoustic observations of zooplankton in lakes using a Doppler current profiler. Freshwater Biology. 2004;49: 1280–1292. 10.1111/j.1365-2427.2004.01267.x

[58] Jiang S, Dickey TD, Steinberg DK, Madin LP. Temporal variability of zooplankton biomass from ADCP backscatter time series data at the Bermuda Testbed Mooring site. Deep Sea Research Part I: Oceanographic Research Papers. 2007;54: 608–636. 10.1016/j.dsr.2006.12.011

[59] H van Haren. Ship-induced effects on bottom-mounted acoustic current meters in shallow seas. Continental Shelf Research. 2009;29: 1809–1814. 10.1016/j.csr.2009.06.002

[60] Rochelle-Newall E, Hulot FD, Janeau JL, Merroune A. CDOM fluorescence as a proxy of DOC concentration in natural waters: a comparison of four contrasting tropical systems. Environ Monit Assess. 2014;186: 589–596. 10.1007/s10661-013-3401-2 24072524

